# Targeting the mevalonate pathway is a novel therapeutic approach to inhibit oncogenic FoxM1 transcription factor in human hepatocellular carcinoma

**DOI:** 10.18632/oncotarget.24781

**Published:** 2018-04-20

**Authors:** Satoshi Ogura, Yuichi Yoshida, Tomohide Kurahashi, Mayumi Egawa, Kunimaro Furuta, Shinichi Kiso, Yoshihiro Kamada, Hayato Hikita, Hidetoshi Eguchi, Hisakazu Ogita, Yuichiro Doki, Masaki Mori, Tomohide Tatsumi, Tetsuo Takehara

**Affiliations:** ^1^ Department of Gastroenterology and Hepatology, Osaka University, Graduate School of Medicine, Suita, Osaka, Japan; ^2^ Department of Molecular Biochemistry and Clinical Investigation, Osaka University, Graduate School of Medicine, Suita, Osaka, Japan; ^3^ Department of Gastroenterological Surgery, Osaka University, Graduate School of Medicine, Suita, Osaka, Japan; ^4^ Division of Molecular Medical Biochemistry, Department of Biochemistry and Molecular Biology, Shiga University of Medical Science, Seta Tsukinowa-cho, Otsu, Shiga, Japan

**Keywords:** cancer metabolism, FOXM1, hepatocellular carcinoma, mevalonate pathway, statin

## Abstract

Dysregulation of cell metabolism is a hallmark of cancer. The mevalonate pathway in lipid metabolism has been implicated as a potential target of cancer therapy for hepatocellular carcinoma (HCC). The role of the Forkhead Box M1 (FoxM1) transcription factor in HCC development has been well documented, however, its involvement in cancer metabolism of HCC has not been fully determined. Here, we hypothesized that FoxM1 is involved in the mevalonate pathway of cholesterol biosynthesis in HCC. Inhibition of the mevalonate pathway by statins, inhibitors of 3-hydroxy-3-methylglutaryl CoA reductase (HMGCR), resulted in reduced expression of FoxM1 and increased cell death in human hepatoma cells. Re-exposure of mevalonate, a product of HMGCR, restored these effects. Likewise, knockdown of HMGCR reduced FoxM1 expression, indicating that FoxM1 expression was regulated by the mevalonate pathway in HCC. Mechanistically, protein geranylgeranylation was found to be responsible for FoxM1 expression and geranylgeranylated proteins, including RhoA, Rac1 or Cdc42, were shown to be involved in this process. In surgically resected human HCC tissues, the gene expression of *FoxM1* had a positive correlation with that of the mevalonate pathway-related genes, such as *HMGCR* or sterol regulatory element-binding protein 2 (*SREBP2*). Furthermore, the gene expression of *FoxM1* along with that of *HMGCR* or *SREBP2* defined prognosis of HCC patients, suggesting the clinical significance of the mevalonate-FoxM1 pathway in human HCC. Our data indicate that FoxM1 links the mevalonate pathway to oncogenic signals in HCC. Thus, we propose a novel therapeutic approach to inhibit FoxM1 by targeting the mevalonate pathway for HCC.

## INTRODUCTION

Hepatocellular carcinoma (HCC) is the one of the most common malignancies in the world [1]. Despite the rapid progress in the development of therapeutic strategies, the prognosis of HCC is still poor due to high rate of recurrence and metastasis [2, 3]. Recently, molecular target agents, such as tyrosine kinases inhibitors, have been developed for the treatment of advanced HCC [4, 5]. To date, however, the efficacy of these drugs seems to be insufficient to achieve a satisfactory therapeutic effect. Thus, it is needed to identify the new pathways based on the detailed molecular mechanisms.

It is well known that metabolic reprogramming occurs in cancer cells and that dysregulation of energy homeostasis may contribute to the development of cancers [6, 7]. In HCC, there is increasing evidence that the mevalonate pathway responsible for cholesterol biosynthesis is implicated in its pathogenesis [8, 9]. Statins are inhibitors of 3-hydroxy-3-methylglutaryl CoA reductase (HMGCR), a rate limiting enzyme for the mevalonate pathway, and are widely used to reduce cholesterol levels, leading to the prevention of cardiovascular diseases [10]. In addition to cholesterol-lowering property, statins have been shown to reduce risk of HCC in clinical studies [11] and have anti-tumor effect on HCC cells in *in vitro* and animal studies [12]. Biologically, the mevalonate pathway is known to play a crucial role for protein prenylation, which is post-translational modification of small GTPases, such as Rho family proteins [13, 14], suggesting that anti-tumor effect of statins on HCC might be through the regulation of protein modification in the mevalonate pathway [15]. However, the precise molecular mechanisms underlying the interplay between the mevalonate pathway and oncogenic signaling in HCC have not been fully determined.

The Forkhead Box M1 (FoxM1) transcription factor, a member of the Fox family of proteins, is highly expressed in a variety of human cancers including HCC [16–19]. Mouse genetic approach have demonstrated that FoxM1 is associated with progression and metastasis of HCC [20–22]. Very recently, we identified FoxM1 expression in tumor tissues as an independent prognostic factor affecting recurrence of HCC and overall survival of HCC patients following surgery, indicating that FoxM1 might not only be a promising therapeutic target but also a prognostic biomarker for HCC [23]. Furthermore, it has been shown that FoxM1 promotes reprograming of glucose metabolism in pancreatic and ovarian cancers [24, 25]. These data suggest that FoxM1 might be implicated in metabolic pathways of HCC pathogenesis. However, the involvement of FoxM1 in cancer lipid metabolism, especially, in the mevalonate pathway of HCC, has not been fully elucidated.

Considering these findings, in this study, we proposed our hypothesis that FoxM1 might be involved in the mevalonate pathway in HCC. To clarify this issue, we utilized *in vitro* culture systems using human hepatoma cell lines along with several inhibitors or metabolites of the mevalonate pathway. Furthermore, we evaluated the gene expression of FoxM1 and that of the mevalonate pathway-related genes in surgically resected HCC tissue samples.

## RESULTS

### FoxM1 expression is regulated by the mevalonate pathway in human hepatoma cells

**Figure 1:**
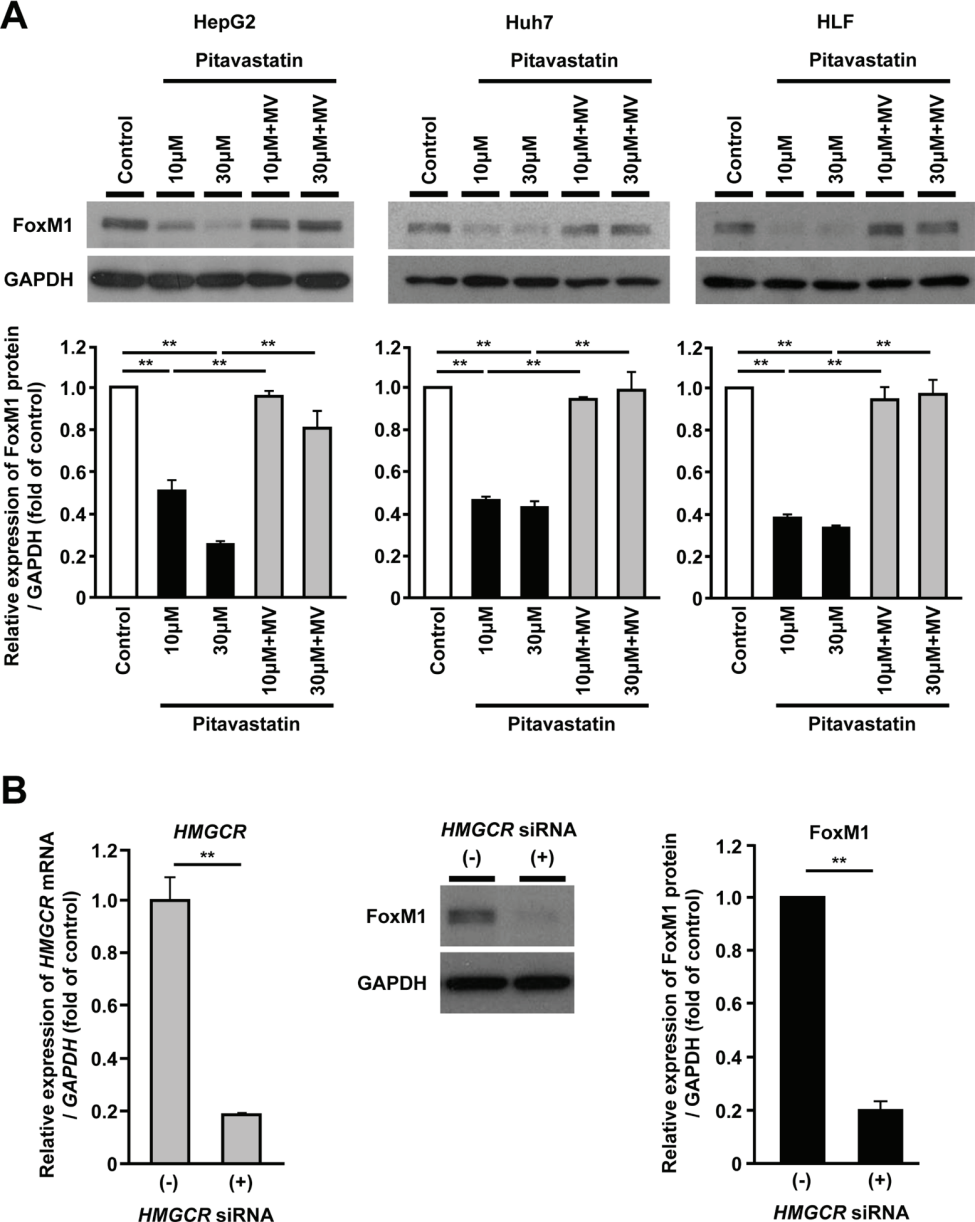
FoxM1 expression is regulated by the mevalonate pathway in human hepatoma cells (**A**) Western blot analysis showing the protein expression of FoxM1 (upper panels) in HepG2 cells (left panel), Huh7 cells (middle panel), and HLF cells (right panel) after treatment with pitavastatin (10 μM or 30 μM), either alone or along with mevalonate (MV, 100 μM), for 24 hours. Quantification of the protein expression of FoxM1 (lower panels) in HepG2 cells (left panel), Huh7 cells (middle panel), and HLF cells (right panel) after treatment with pitavastatin (10 μM or 30 μM), either alone or along with MV (100 μM), for 24 hours. DMSO was used as control. (**B**) Effect of siRNA-mediated depletion of *HMGCR* on FoxM1 protein expression in HepG2 cells. Quantification of *HMGCR* gene expression in siRNA against *HMGCR*-treated HepG2 cells (left panel). Western blot analysis showing the protein expression of FoxM1 in siRNA against *HMGCR*-treated HepG2 cells (representative images: middle panel, quantification of the protein expression: right panel). The values of the protein expression were normalized to the control. Data are expressed as mean ± SEM, ^**^p < 0.01.

To investigate the involvement of FoxM1 in the mevalonate pathway, we examined whether the inhibition of the mevalonate pathway might affect the FoxM1 protein expression in human hepatoma cells. HepG2, Huh7 and HLF cells were treated with pitavastatin, a synthetic HMGCR inhibitor, and the FoxM1 protein expression was examined using Western blot analysis. Administration of pitavastatin significantly reduced the FoxM1 protein expression in a dose-dependent manner in these hepatoma cell lines (Figure 1A). Furthermore, re-exposure to mevalonate (MV), a product of HMGCR, restored the reduction of FoxM1 protein expression induced by pitavastatin (Figure 1A). The effect of pitavastatin or MV on *FoxM1* expression was also confirmed by the quantitative real-time RT-PCR analysis (Supplementary Figure 1). Similar results were obtained when HepG2 cells were treated with simvastatin or fluvastatin (Supplementary Figure 2A and 2C). Moreover, siRNA-mediated depletion of *HMGCR* caused the reduction of the FoxM1 protein expression in HepG2 cells (Figure 1B). These results indicated that FoxM1 was regulated by mevalonate pathway in human hepatoma cells.

### Inhibition of HMGCR decreases the nuclear expression and the transcriptional activity of FoxM1 in human hepatoma cells

**Figure 2:**
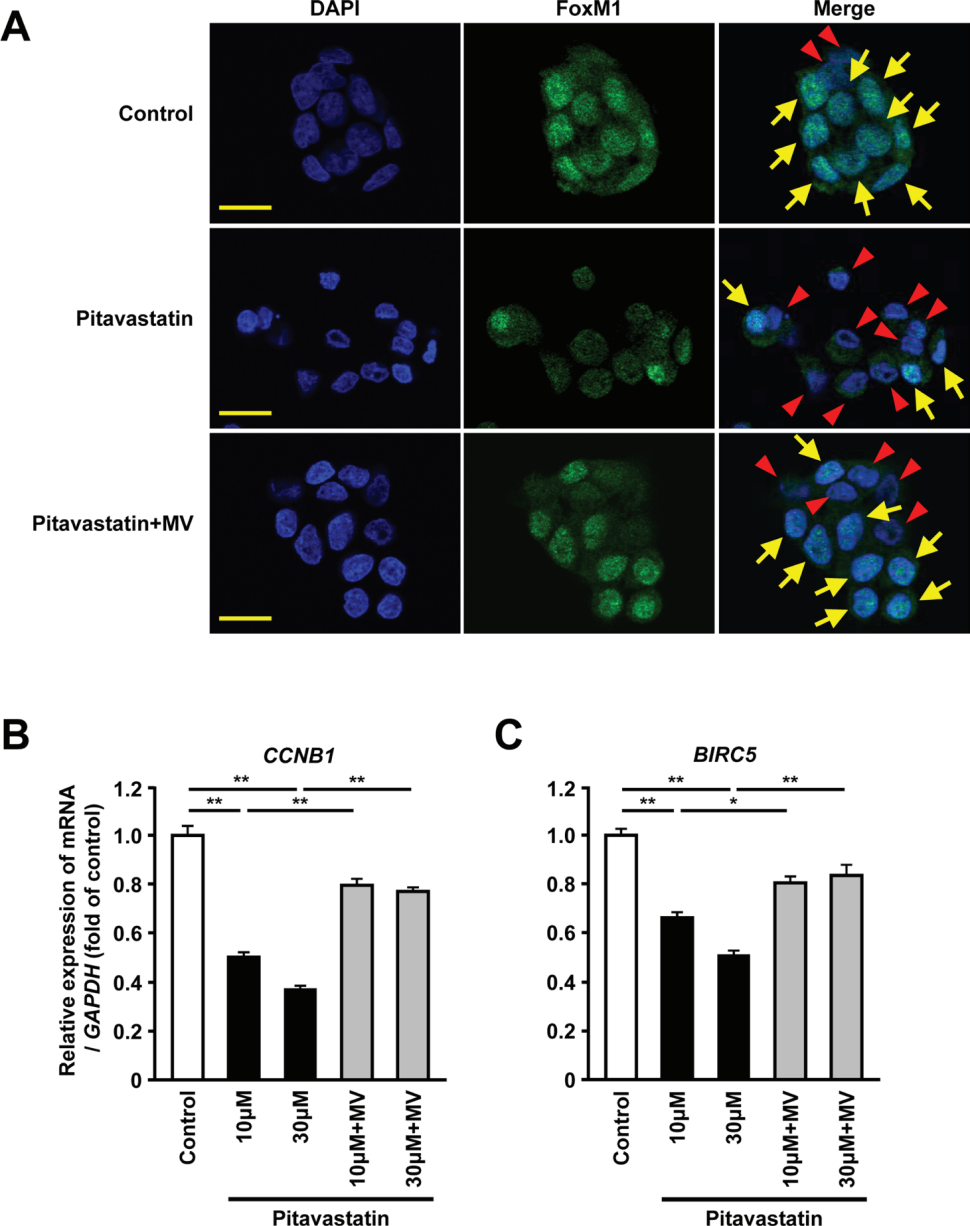
Inhibition of HMGCR decreases the nuclear expression and the transcriptional activity of FoxM1 in human hepatoma cells (**A**) Immunofluorescence analysis showing the protein expression of FoxM1 (green) in HepG2 cells treated with pitavastatin (10 μM) or pitavastatin (10 μM) plus MV (100 μM) for 24 hours. Nuclei were stained by DAPI (blue). Scale bar: 20 μm. Arrows or arrowheads indicate the cell with or without the nuclear FoxM1 protein expression, respectively. (**B–C**) Quantitative real-time RT-PCR analysis showing the gene expressions of *CCNB1* (B) or *BIRC5* (C) in HepG2 cells after treatment with pitavastatin (10 μM or 30 μM), either alone or along with MV (100 μM) for 24 hours. DMSO was used as control. Data are expressed as mean ± SEM, ^*^p < 0.05, ^**^p < 0.01.

To confirm the regulation of FoxM1 expression via the mevalonate pathway, we immunohistochemically examined the expression of FoxM1 in HepG2 cells treated with pitavastatin. Immunofluorescence analysis showed that the administration of pitavastatin reduced nuclear expression of FoxM1 whereas its expression was detected mainly in the nucleus in control cells. Furthermore, re-exposure to MV restored the nuclear expression of FoxM1 (Figure 2A). Because the nuclear expression of FoxM1 is shown to be required for its transcriptional activity [26], we also examined whether inhibition of HMGCR might affect expression of FoxM1 target genes. The administration of pitavastatin resulted in a significant reduction of *CCNB1* or *BIRC5,* known FoxM1 target genes [27, 28]. Re-exposure to MV restored the expression of these genes (Figure 2B and 2C). Together, these data suggested that the mevalonate pathway could regulate the transcriptional activity of FoxM1 in human hepatoma cells.

### Reduced expression of FoxM1 by HMGCR-inhibition is associated with increased cell death in human hepatoma cells

**Figure 3:**
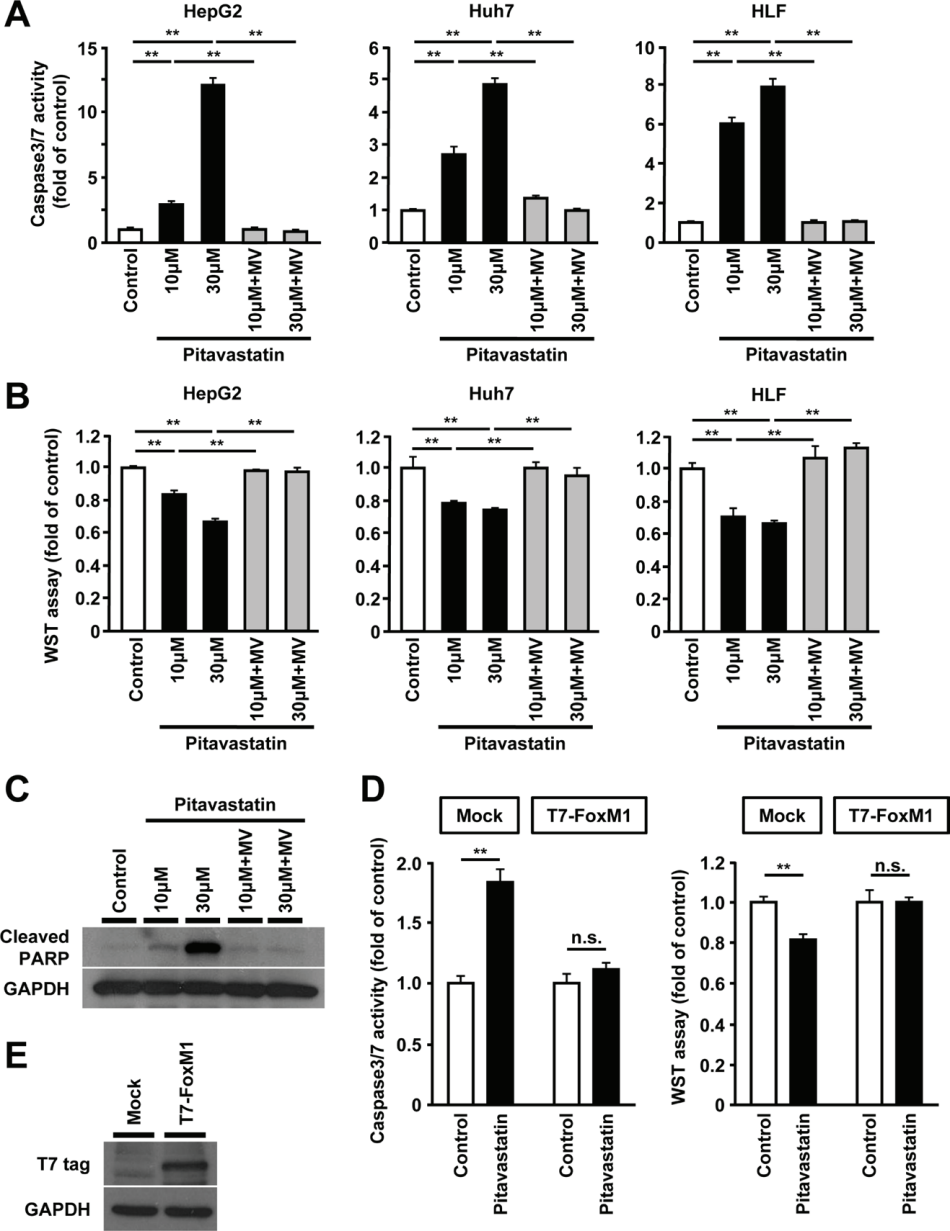
Reduced expression of FoxM1 by HMGCR-inhibition is associated with increased cell death in human hepatoma cells (**A**) Assessment of cell death by caspase 3/7 activity in HepG2 cells (left panel), Huh7 cells (middle panel), and HLF cells (right panel) after treatment with pitavastatin (10 μM or 30 μM), either alone or along with MV (100 μM) for 48 hours. (**B**) Assessment of cell viability by WST assay in HepG2 cells (left panel), Huh7 cells (middle panel), and HLF cells (right panel) after treatment with pitavastatin (10 μM or 30 μM), either alone or along with MV (100 μM) for 48 hours. (**C**) Western blot analysis showing the protein expression of cleaved PARP in HepG2 cells after treatment with pitavastatin (10 μM or 30 μM), either alone or along with MV (100 μM) for 24 hours. (**D**) Effect of overexpression of FoxM1 on cell death or viability in HepG2 cells treated with pitavastatin (10 μM). (**E**) Western blot analysis showing the protein expression of T7-tagged FoxM1 (T7-FoxM1) using anti-T7 antibody. DMSO was used as control (for A, B, C, and D). Data are expressed as mean ± SEM, ^*^p < 0.05, ^**^p < 0.01, n.s. not significant.

Inhibition of HMGCR is shown to result in increased cell death in several cancer cells [14]. Therefore, we examined the effect of statin on cell death of human hepatoma cells. Consistent with previous reports, administration of pitavastatin induced an increase of cell death and re-exposure to MV restored these effects in HepG2, Huh7 and HLF cells (Figure 3A and 3B). Effect of pitavastatin on cell death was confirmed by the expression of cleaved PARP (Figure 3C). Furthermore, similar results were observed in case of other types of statins, such as simvastatin or fluvastatin (Supplementary Figure 2B and 2D). Furthermore, overexpression of FoxM1 resulted in loss of statin-induced cell death (Figure 3D and 3E). Collectively, these results suggest that the cell death induced by the inhibition of HMGCR might occur via FoxM1.

### *FoxM1* expression is regulated via protein geranylgeranylation in human hepatoma cells

**Figure 4:**
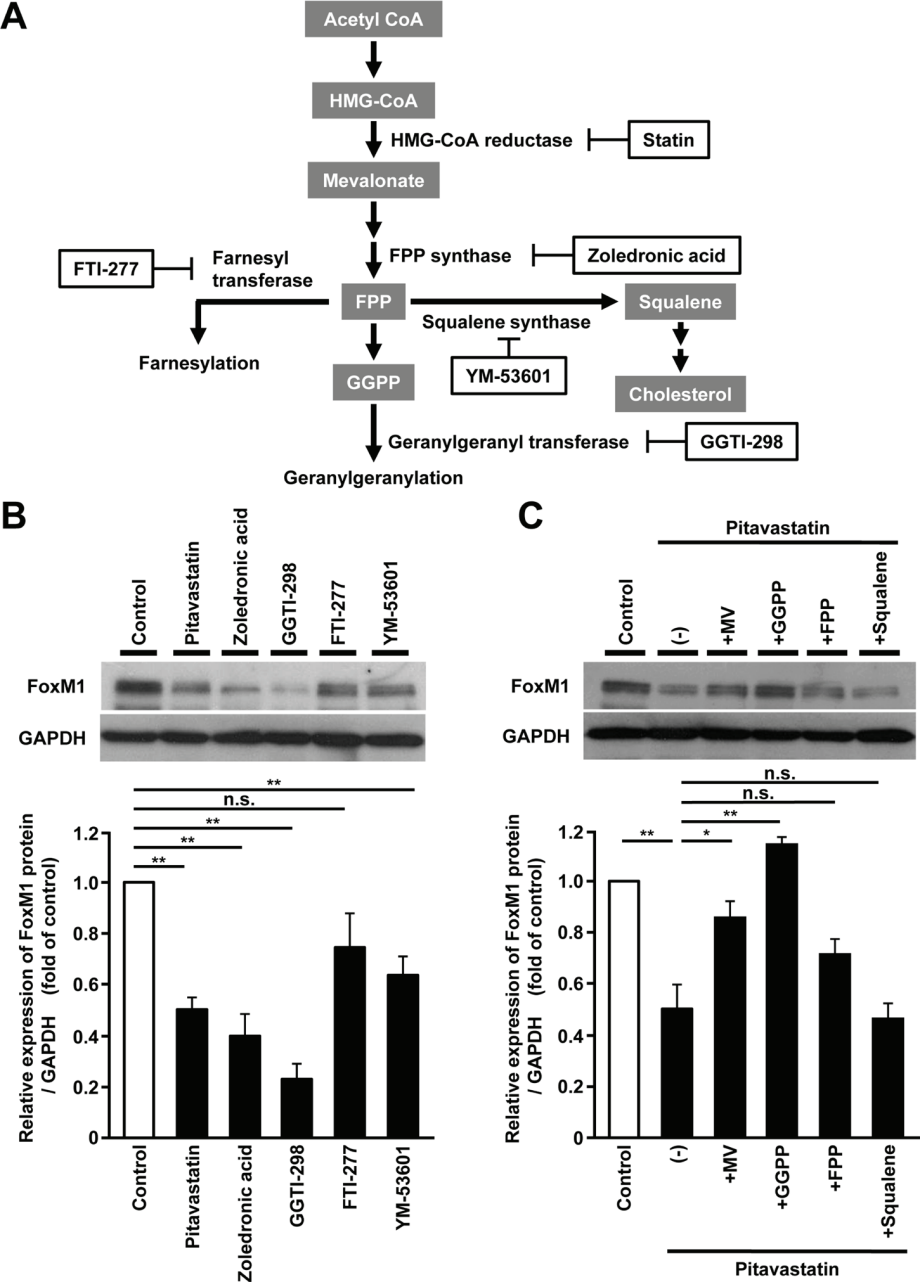
FoxM1 expression is regulated via protein geranylgeranylation in human hepatoma cells (**A**) Schematic overview of the mevalonate pathway. The inhibitors or the products for the mevalonate pathway are shown in a frame or in a gray box, respectively. (**B**) Western blot analysis showing the protein expression of FoxM1 in HepG2 cells treated with the inhibitors for the mevalonate pathway, including pitavastatin (10 μM), zoledronic acid (200 μM), GGTI-298 (10 μM), FTI-277 (10 μM), or YM-53601 (5 μM), for 24 hours (upper panel). Quantification of the protein expression of FoxM1 in HepG2 cells treated with these inhibitors (lower panel). (**C**) Western blot analysis showing the protein expression of FoxM1 in HepG2 cells treated with pitavastatin (10 μM), either alone or along with the products for the mevalonate pathway, including MV (100 μM), geranylgeranyl pyrophosphate (GGPP, 10 μM), farnesyl pyrophosphate (FPP, 10 μM), or squalene (10 μM) for 24 hours (upper panel). Quantification of the protein expression of FoxM1 in HepG2 cells treated with pitavastatin (10 μM) alone or along with these products (lower panel). The values of the protein expression were normalized to the control. Data are expressed as mean ± SEM, ^*^p < 0.05, ^**^p < 0.01, n.s. not significant.

To investigate the specific metabolic intermediates responsible for the regulation of FoxM1 expression, we used several inhibitors of enzymes or products in the mevalonate pathway [29] (Figure 4A). Farnesyl pyrophosphate (FPP) synthase inhibitor (zoledronic acid), or geranylgeranyl transferase inhibitor (GGTI-298), had almost the same effect as pitavastatin on FoxM1 expression, whereas farnesyl transferase inhibitor (FTI-277) or squalene synthase inhibitor (YM-53601) had less effect (Figure 4B). Re-exposure to geranylgeranyl pyrophosphate (GGPP) restored pitavastatin-induced reduction of FoxM1 expression, whereas FPP or squalene had less effect (Figure 4C). Taken together, these results indicated that the regulation of FoxM1 expression in the mevalonate pathway might be mainly through protein geranylgeranylation in human hepatoma cells.

### Rho family proteins are involved in the mevalonate pathway-dependent regulation of FoxM1

**Figure 5:**
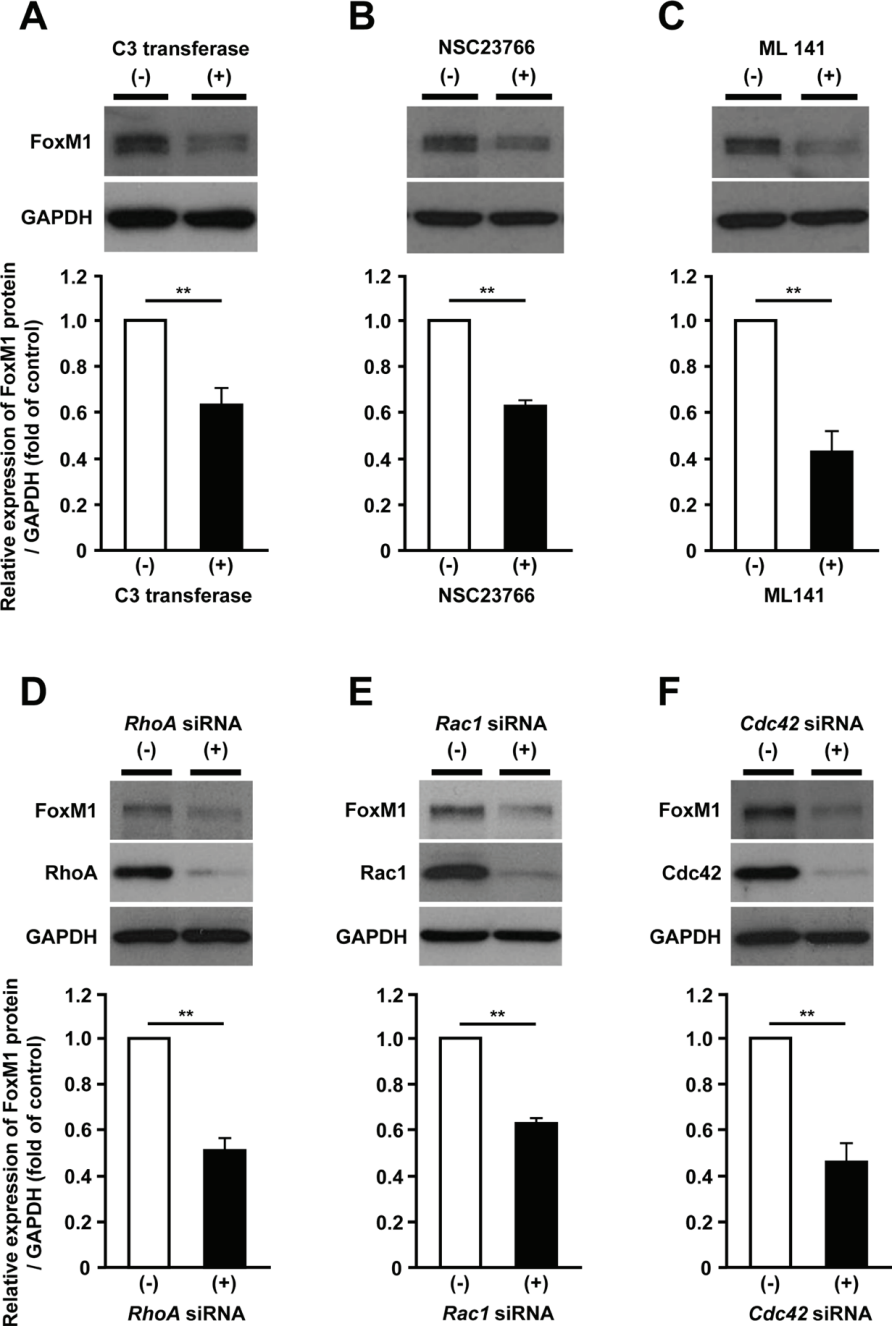
Rho family proteins are involved in the mevalonate pathway-dependent regulation of FoxM1 (**A–C**) Western blot analysis showing the protein expression of FoxM1 in HepG2 cells treated with 2 μg/ml of RhoA inhibitor C3 transferase (A, upper panel), 100 μM of Rac1 inhibitor NSC23766 (B, upper panel), or 20 μM of Cdc42 inhibitor ML141 (C, upper panel). Quantification of the protein expression of FoxM1 in HepG2 cells with these inhibitors (lower panels). (**D–F**) Western blot analysis showing the protein expression of FoxM1 transfected with siRNA against *RhoA* (D, upper panel), *Rac1* (E, upper panel) or *Cdc42* (F, upper panel). Quantification of the protein expression of FoxM1 in HepG2 cells transfected with these siRNAs (lower panels). The values of the protein expression were normalized to the control. Data are expressed as mean ± SEM, ^**^p < 0.01.

To investigate which specific factors regulate FoxM1 expression in the mevalonate pathway, we focused on the Rho family proteins as they require geranylgeranylation when they are activated [13]. To explore the requirement of Rho family proteins in regulating FoxM1 expression, we used the inhibitors for major Rho family proteins, such as RhoA, Rac1 and Cdc42. Administration of each inhibitor significantly reduced FoxM1 expression (Figure 5A–5C). Additionally, siRNA-mediated depletion of *RhoA*, *Rac1* and *Cdc42* also reduced FoxM1 expression (Figure 5D–5F). These results suggested that the mevalonate pathway-dependent FoxM1 expression could be regulated via Rho family proteins.

### FoxM1 is associated with the mevalonate pathway in HCC patients

**Figure 6:**
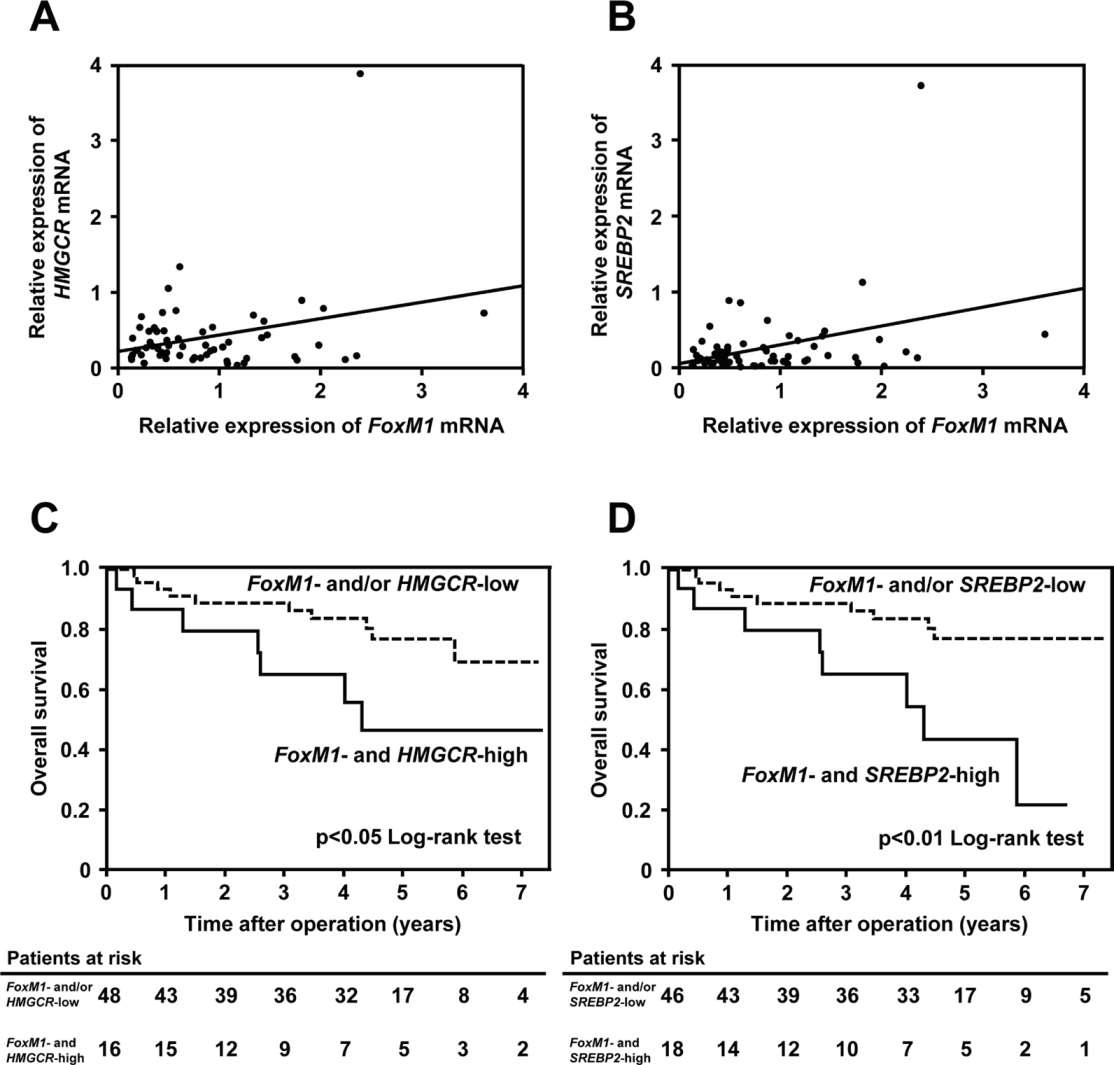
FoxM1 is associated with the mevalonate pathway in HCC patients (**A**) Correlation between the gene expression of *FoxM1* and *HMGCR* in tumor tissues of HCC patients (*r* = 0.29, *p <* 0.05). (**B**) Correlation between the gene expression of *FoxM1* and *SREBP2* in tumor tissues of HCC patients (*r* = 0.35, *p <* 0.01). (**C** and **D**) The relationship between the overall survival of *FoxM1* and *HMGCR* (C)/*SREBP2* (D) expression in human HCC. High group is defined as higher gene expression than median.

To confirm the clinical significance regarding the mevalonate pathway-dependent regulation of FoxM1 in human HCC, we finally examined the expressions of *FoxM1* and the mevalonate pathway-related genes in tumor tissues of HCC patients, whose clinicopathological features are summarized in Table 1. The quantitative real-time RT-PCR analysis showed that the expression of *FoxM1* had a significant positive correlation with that of *HMGCR*, a major enzyme of the mevalonate pathway, in these tissues (Figure 6A, *r* = 0.29, *p <* 0.05). This analysis also revealed a positive correlation between the expression of *FoxM1* and that of sterol regulatory element-binding protein 2 (*SREBP2*), a main transcriptional factor which regulates *HMGCR* expression (Figure 6B, *r* = 0.35, *p <* 0.01). The overall survival of *FoxM1*- and *HMGCR*-high group was found to be significantly lower than that of *FoxM1*- and/or *HMGCR*-low group (Figure 6C). Likewise, the overall survival of *FoxM1*- and *SREBP2*-high group was found to be significantly lower than that of *FoxM1*- and/or *SREBP2*-low group (Figure 6D). As shown in Supplementary Tables 1 and 2, *FoxM1*- and *HMGCR*-high group had a statistically significant lower rate of liver cirrhosis of liver histology than *FoxM1*- and/or *HMGCR*-low group (*p* = 0.0337) and *FoxM1*- and *SREBP2*-high group had a statistically significant higher PIVKA-II level than *FoxM1*- and/or *SREBP2*-low group (*p* = 0.0294). Furthermore, as shown in Supplementary Tables 3 and 4, multivariate analysis showed that *FoxM1*- and *SREBP2*-high expression in tumor tissues was identified as an independent prognostic factor of overall survival (hazard ratio: 3.24; 95% confidence interval: 1.18-8.89; *p* = 0.0238). Collectively, these results would suggest the clinical implications of the mevalonate-FoxM1 pathway in human HCC.

**Table 1:** Clinicopathological features of HCC patients

	*n* = 64
**Age (y.o.), mean (range)**	64.2 (36–84)
**Gender : Male/Female**	57/7
**HBs-Ag : Negative/Positive**	49/15
**HCV-Ab : Negative/Positive**	31/33
**Child-Pugh score : A/B**	57/7
**AFP (ng/ml), median (range)**	20.5 (3–390000)
**PIVKA-II (mAU/ml), median (range)**	308 (13–361200)
**Liver histology : NL/CH/LC**	7/40/17
**Maximum tumor size (mm), mean (range)**	44.4 (7–320)

NL, normal liver; CH, chronic hepatitis; LC: liver cirrhosis

## DISCUSSION

Evidence has accumulated regarding the crucial role of FoxM1 in cancer development and progression [18, 23, 30], suggesting the possible application of FoxM1 as a therapeutic target against cancer. In this study, for the first time, we have shown that FoxM1 acts as a downstream target for the mevalonate pathway of cholesterol biosynthesis in human HCC. Our *in vitro* findings showed that statins, well-known HMGCR inhibitors, reduced FoxM1 expression, indicating that statins could be novel inhibitors to reduce FoxM1 in HCC. We also demonstrated that protein geranylgeranylation was responsible for the regulation of FoxM1 expression in the mevalonate pathway. Using clinical samples, we observed a significant positive correlation between the gene expression of *FoxM1* and that of *HMGCR*, a rate limiting enzyme for mevalonate pathway, or *SREBP2*, a master regulator of the mevalonate pathway [14], in HCC tissues. Furthermore, the expression of *FoxM1* along with that of *HMGCR* or *SREBP2* was found to define the prognosis of HCC patients. Therefore, we propose that the regulation of FoxM1 via the mevalonate pathway may open new avenues for the development of molecular targeted therapies against HCC.

FoxM1 is a well-defined transcription factor which is crucial for cell proliferation and cell cycle progression [31, 32]. Although FoxM1 is rarely expressed in quiescent or differentiated cells, its expression is highly elevated in proliferating cells or a variety of cancers [16, 17, 33]. In cancer cells, FoxM1 is shown to act downstream of growth signals, such as Ras-mitogen-activated protein kinase pathway or phosphatidylinositol 3-kinase-AKT pathway [34, 35]. Likewise, loss of tumor suppressor, such as p53 or p19Arf, is shown to control FoxM1 expression in cancer cells [20, 21, 36], suggesting that FoxM1 is involved in multiple hallmarks of cancer [37]. However, the links between FoxM1 and cancer metabolism has been yet fully investigated. Recently, it has been shown that FoxM1 is involved in glucose metabolism of pancreatic or ovarian cancers [24, 25]. Nevertheless, to date, it has remained unclear whether FoxM1 is involved in the mevalonate pathway of cholesterol biosynthesis, which is known to play a crucial role in lipid metabolism in cancer. In this study, we showed that the expression of *FoxM1* is correlated with that of *HMGCR* or *SREBP2* in tumor tissues of HCC patients. Our findings have raised the possibility that FoxM1 would link the mevalonate pathway of cholesterol biosynthesis to the oncogenic signals in HCC.

The mevalonate pathway is a major metabolic route to convert acetyl-CoA to cholesterol that is essential for cell proliferation [13, 14]. Dysregulation of the mevalonate pathway is known to promote oncogenesis in several cancers [38]. So far, several lines of evidence have shown that HMGCR has a key role in the oncogenic potential of the mevalonate pathway [14, 38]. In this study, inhibition of HMGCR by statins resulted in not only increased cell death but also the reduced expression of FoxM1 in human hepatoma cells. In addition, forced expression of FoxM1 restored statins-mediated cell death of human hepatoma cells, indicating that cell death in the mevalonate pathway might be regulated through FoxM1. This mevalonate pathway-mediated regulation of FoxM1 was confirmed by the expression of its known target genes, such as *CCNB1* or *BIRC5*. The data also suggest that the additional treatment of MV did not seem to rescue the expression of these target genes completely. One possible explanation for this discrepancy is that other factors other than FoxM1, such as p53, might be involved in the regulation of *CCNB1* or *BIRC5* in the mevalonate pathway [39, 40]. Collectively, our data suggest that FoxM1 is a downstream target of the mevalonate pathway in HCC cells.

The accumulation of intermediate metabolites in the mevalonate pathway induces post-translational modification of signaling proteins that is required for cell proliferation, survival, and migration [13, 14]. This modification is referred to as prenylation, including protein geranylgeranylation and protein farnesylation [13, 14]. In this study, we showed that protein geranylgeranylation rather than protein farnesylation is required for the regulation of FoxM1 expression in the mevalonate pathway. We further identified Rho-family of small GTPases, RhoA, Rac1 and Cdc42, as geranylgeranylated proteins that regulate FoxM1 expression. Furthermore, as shown in Supplementary Figure 3, we found that the knockdown of *FoxM1*, *HMGCR* or *Cdc42* caused a statistically significant increase in cell death, whereas the knockdown of *RhoA* or *Rac1* did not. Our results showing the induction of cell death in HMGCR- or FoxM1-depleted HepG2 cells would be consistent with the results of statins treatment, suggesting the possibility that cell death is indeed by a similar mechanism in both cases. Although RhoA, Rac1 and Cdc42, were associated with the regulation of FoxM1 expression, the induction of cell death was observed in Cdc42-depleted HepG2 cells but not in RhoA- or Rac1-depleted HepG2 cells. One possible explanation for this discrepancy might be that the role of these Rho family proteins in the downstream of the mevalonate-FoxM1 pathway might differ in cell death of human hepatoma cells. At this stage, the detailed molecular mechanisms by which RhoA, Rac1 and Cdc42 coordinate FoxM1 expression in HCC cells has remained unclear. Further investigation will be needed to elucidate this issue.

The liver X receptors (LXRs), such as LXRα or LXRβ, are known to regulate the uptake, transport, and efflux of cholesterol in the liver and macrophages [41]. There have been several evidences showing the involvement of LXRs in a variety of cancers including HCC [42]. A previous report showed that the activation of LXRα resulted in the downregulation of FoxM1 and the suppression of proliferation in human hepatoma cells [43]. As shown in Supplementary Figure 4, the administration of pitavastatin significantly increased the gene expression of *LXRα* in HepG2 cells and re-exposure to mevalonate restored the increase of *LXRα* gene expression induced by pitavastatin. These data indicate that the up-regulation of *LXRα* by statin might be one possible mechanism underlying the inhibition of FoxM1 expression by statin. However, at this stage, the relation between the mevalonate pathway and the LXRα-mediated pathway in the regulation of FoxM1 has remained unclear. Further investigation will be needed to understand detailed mechanisms.

So far, several therapeutic strategies have been proposed to target FoxM1 in cancer. In mouse models, a cell-penetrating ARF peptide that reduces FoxM1 transcriptional activity has been shown to be effective to treat HCC [20, 21]. A high-throughput screening identified a thiazole antibiotics Syomycin A as a potent FoxM1 inhibitor [44] and thiostrepton, a similar type of thiazole antibiotics, has been shown to inhibit FoxM1 transcriptional activity by direct binding to FoxM1 in several human cancer cells including HCC cells [45]. Likewise, another screening also identified a small molecule FDI-6 as a potent FoxM1 inhibitor in breast cancer cells [46]. Despite these findings, no drugs targeting FoxM1 are available in clinical use for cancer therapy so far. In this study, we identified statins, FDA-approved cholesterol-lowering drugs, as novel FoxM1 inhibitors against HCC. In this study, we used various pharmaceutical inhibitors for the mevalonate pathway and showed that zoledronic acid or GGTI-298 had a potential to inhibit FoxM1 expression same as statins. Previous reports showed the possible toxic effects of these pharmaceutical inhibitors which we used in our study; pitavastatin may exhibit myotoxicity including myopathy, myalgia, myositis, or rhabdomyolysis [47]; zoledronic acid may induce hypocalcaemia, secondary hyperparathyroidism, or renal toxicity [48]. Because other inhibitors, such as GGTI-298 [49], FTI-277 [50], or YM-53601 [51], are still in preclinical stages, toxic effects in clinical use remain unclear. Collectively, it is worth noting that drugs already available in the clinical practice have an inhibitory effect on FoxM1 expression in HCC.

In conclusion, in this study, we demonstrated that oncogenic FoxM1 transcription factor functions downstream of the mevalonate pathway in HCC. Our findings also provided new insights on the interplay between the mevalonate pathway and oncogenic signals. Thus, the inhibition of FoxM1 by targeting the mevalonate pathway would be a novel therapeutic option for the treatment of HCC.

## MATERIALS AND METHODS

### Reagents

Pitavastatin was a gift from Kowa Pharmaceutical Co., Ltd. (Tokyo, Japan). Simvastatin, fluvastatin sodium hydrate, mevalonolactone, GGTI-298 trifluoroacetate salt hydrate, FTI-277 trifluoroacetate salt, zoledronic acid monohydrate, farnesyl pyrophosphate ammonium salt, geranylgeranyl pyrophosphate ammonium salt, squalene, NSC23766 trihydrochloride, and ML141 were obtained from Sigma-Aldrich (Saint Louis, MO, USA). YM-53601 was obtained from Cayman Chemical Co. (Ann Arbor, MI, USA). C3 transferase was obtained from Cytoskeleton, Inc. (Denver, CO, USA).

### Cell culture

HepG2, Huh7 and HLF cells were obtained from the Japanese Cancer Resources Bank (JCRB, Tokyo, Japan) between 2011 and 2015 and have been cryopreserved in liquid nitrogen until use. Cells were used within 10 passages after thawing and were cultured in DMEM (Sigma-Aldrich, Saint Louis, MO, USA) supplemented with 10% FCS and Antibiotic-Antimycotic (Thermo Fisher Scientific, Inc., Waltham, MA, USA) in an atmosphere of 5% CO_2_ in air at 37° C. Test for mycoplasmal contamination was performed using MycoAlert mycoplasma detection kit (Lonza, Walkersville, MD, USA). Cells were seeded on 6-well plate at 1.0 × 10^5^ cells/well. After an overnight culture, cells were treated with reagents for 24 hours. Cells were then harvested to prepare protein extracts.

### Western blot analysis

Protein extraction and Western blot analysis were performed as previously described [52]. The following primary antibodies were used: FoxM1 (D12D5), Cleaved PARP (Asp214) (D64E10), RhoA (67B9), Rac1/2/3, Cdc42 (11A11) (Cell Signaling Technology, Inc., Danvers, MA, USA), T7-tag monoclonal antibody (Merck KGaA, Darmstadt, Germany), GAPDH (Trevigen, Inc., Gaithersburg, MD, USA). Donkey anti-rabbit IgG-HRP (Santa Cruz Biotechnology, Inc., Santa Cruz, CA, USA) and anti-mouse IgG-HRP (Promega Corp., Madison, WI, USA) were used as a secondary antibody.

### Quantitative real-time RT-PCR

Cells were seeded on 6-well plate at 1.0 × 10^5^ cells/well. After an overnight culture, cells were treated with reagents for 24 hours. Total RNA was extracted and purified using QIAshredder (Qiagen, Hilden, Germany) and RNeasy Mini Kit (Qiagen, Hilden, Germany) according to the manufacturer's instructions as previously described [52]. cDNA was reverse-transcribed from 1μg of total RNA using ReverTra Ace qPCR RT Master Mix (Toyobo Co., Ltd., Osaka, Japan). Quantitative real-time RT-PCR was performed with QuantiTect Primer Assays (*FoxM1*: QT00000140, *HMGCR*: QT00004081, *CCNB1*: QT00006615, *BIRC5*: QT01679664, *SREBP2*: QT00052052, *RhoA*: QT00044723, *Rac1*: QT00065856, *Cdc42*: QT00066528, *LXRα (NR1H3)*: QT00065156, *GAPDH*: QT00079247, Qiagen, Hilden, Germany), using Light Cycler 1.5 (Roche Diagnostics, Basel, Switzerland) and Quant Studio 6 (Thermo Fisher Scientific, Inc., Waltham, MA, USA). Relative quantitation of gene expression was analyzed with ΔΔCt method, using *GAPDH* as an internal control.

### siRNA transfection

Stealth RNAi siRNA of *FoxM1* (HSS177135), *HMGCR* (HSS104864), *RhoA* (VHS40471), *Rac1* (VHS40447) and *Cdc42* (VHS40393) were obtained from Thermo Fisher Scientific, Inc. (Waltham, MA, USA). 20 nM of gene specific siRNA or Stealth RNAi siRNA Negative Control (Thermo Fisher Scientific, Inc., Waltham, MA, USA) was transfected with Lipofectamine RNAiMAX Transfection Reagent (Thermo Fisher Scientific, Inc., Waltham, MA, USA) after incubating in Opti-MEM I Reduced serum medium (Thermo Fisher Scientific, Inc., Waltham, MA, USA) as previously described [52].

### Immunofluorescent staining

Cells were seeded on poly-L-lysine coated culture cover glass (13 mm, Matsunami Glass Industry, Ltd., Osaka, Japan) placed in 24-well plate at 3.0 × 10^4^ cells/well. After an overnight culture, cells were treated with reagents for 24 hours. Cells were fixed with 4% paraformaldehyde for 20 minutes at room temperature. After permeabilized with 0.5% Triton-X for 20 minutes at room temperature, the cells were treated with 3% bovine serum albumin (Sigma-Aldrich, Saint Louis, MO, USA) for 30 minutes at room temperature. After this blocking procedure, cells were treated with a primary antibody for overnight at 4° C, and then a secondary antibody for 1 hour at room temperature. We used the following antibodies: FoxM1 (D12D5, Cell Signaling Technology, Inc., Danvers, MA, USA), Goat anti-rabbit IgG (H+L) Secondary Antibody, Alexa Flour 488 conjugate (Thermo Fisher Scientific, Inc., Waltham, MA, USA). Cover glass was mounted with DAPI Fluoromount-G (Southern Biotechnology Associates, Inc., Birmingham, AL, USA) on slide glass.

### Assessment of caspase3/7 activity

Cells were seeded on 96-well plate at 3.5 × 10^3^ cells/well. After an overnight culture, cells were treated with reagents for 24 or 48 hours. The caspase 3/7 activity of supernatant was assessed with Caspase-Glo 3/7 Assay (Promega Corp., Madison, WI, USA), according to the manufacturer's instructions.

### WST assay

Cells were seeded on 96-well plate at 3.5 × 10^3^ cells/well. After an overnight culture, cells were treated with reagents for 48 hours. The WST assay was assessed with Cell Count Reagent SF (Nacalai Tesque, Inc., Kyoto, Japan), according to the manufacturer's instructions.

### Plasmid transfection

T7-tagged FoxM1 plasmid (T7-FoxM1) was provided by Dr. Pradip Raychaudhuri [34]. After seeding cells and overnight culture, the plasmid was transfected with FuGENE HD Transfection Reagent (Promega Corp., Madison, WI, USA), according to the manufacturer's instructions.

### Patients

We examined 64 patients undergoing curative hepatic resection for HCC at the Department of Surgery, Osaka University Hospital from May 2001 to November 2011. None of the patients had undergone transcatheter arterial embolization or transcatheter arterial chemoembolization before surgery. This study was performed according to the ethical guidelines of the Declaration of Helsinki and was approval to use the resected samples from the Institutional Review Board (IRB) Committees at Osaka University (IRB No. 13556 and 15267), and informed consent was obtained from all patients. In this study, the “high” group is defined as the patients with higher gene expression than median; the “low” group is defined as those with lower gene expression than median.

### Statistical analysis

Statistical analysis was performed with JMP Pro 12.2.0 (SAS Institute, Inc., Cary, NC, USA) using Student's *t*-test. Data was represented as mean ± standard error of the mean (SEM) from three biological replicates. We used Pearson's correlation coefficients to determine the correlation between the genes expression in human HCC. Overall survival was calculated using Kaplan–Meier method and compared by Log-rank test. Patients’ characteristics were compared using Mann–Whitney *U* test or Pearson's chi-square test. The prognostic factors were identified using the Cox proportional hazards model; the factors chosen using simple Cox regression were further examined using multiple Cox regression. Statistical significance was defined as *p* < 0.05.

## SUPPLEMENTARY MATERIALS FIGURES AND TABLES



## Abbreviations

FoxM1: Forkhead Box M1 transcription factor
FPP: farnesyl pyrophosphate
GAPDH: glyceraldehyde-3-phosphate dehydrogenase
GGPP: geranylgeranyl pyrophosphate
HCC: hepatocellular carcinoma
HMGCR: 3-hydroxy-3-methylglutaryl CoA reductase
RT-PCR: reverse-transcription polymerase chain reaction
siRNA: small-interfering RNA
SREBP2: sterol regulatory element-binding protein 2.
