# Supplementary material for: Targeting the mevalonate pathway is a novel therapeutic approach to inhibit oncogenic FoxM1 transcription factor in human hepatocellular carcinoma
Source: Oncotarget. 2018 Apr 20;9(30):21022–35. doi: 10.18632/oncotarget.24781 (PMC5940385; doi:10.18632/oncotarget.24781)
Supplement: Supplementary file 1 [file oncotarget-09-21022-s001.pdf]

Targeting the mevalonate pathway is a novel therapeutic approach to inhibit oncogenic FoxM1 transcription factor in human hepatocellular carcinoma

SUPPLEMENTARY MATERIALS

SUPPLEMENTARY MATERIAL AND METHODS

Reagents

Pitavastatin was a gift from Kowa Pharmaceutical Co., Ltd. (Tokyo, Japan). Simvastatin, fluvastatin sodium hydrate, and mevalonolactone were obtained from Sigma-Aldrich (Saint Louis, MO, USA).

siRNA transfection

Stealth RNAi siRNA of *FoxM1* (HSS177135), *HMGCR* (HSS104864), *RhoA* (VHS40471), *Rac1* (VHS40447) and *Cdc42* (VHS40393) were obtained from Thermo Fisher Scientific, Inc. (Waltham, MA, USA). siRNA transfection was performed as described in the text.

Quantitative real-time RT-PCR

Quantitative real-time RT-PCR was performed with QuantiTect Primer Assays (*FoxM1*: QT00000140, *HMGCR*: QT00004081, *RhoA*: QT00044723, *Rac1*: QT00065856, *Cdc42*: QT00066528, *LXRα (NR1H3)*: QT00065156, *GAPDH*: QT00079247, Qiagen, Hilden, Germany), using Light Cycler 1.5 (Roche Diagnostics, Basel, Switzerland) and Quant Studio 6 (Thermo Fisher Scientific, Inc., Waltham, MA, USA). Relative quantitation of gene expression was analyzed with ΔΔCt method, using *GAPDH* as an internal control.

Assessment of caspase3/7 activity, WST assay, and Western blot analysis

The caspase3/7 activity was assessed with Caspase-Glo 3/7 Assay (Promega Corp., Madison, WI, USA). The WST assay was assessed with Cell Count Reagent SF (Nacalai Tesque, Inc., Kyoto, Japan). Western blot analysis was performed as described in the text.

Statistical analysis

Statistical analysis was performed with JMP Pro 12.2.0 (SAS Institute, Inc., Cary, NC, USA) using Student's *t*-test. Data was represented as mean ± standard error of the mean (SEM) from three biological replicates. Patients' characteristics were compared using Mann–Whitney *U* test or Pearson's chi-square test. The prognostic factors were identified using the Cox proportional hazards model; the factors chosen using simple Cox regression were further examined using multiple Cox regression. Statistical significance was defined as $p < 0.05$.

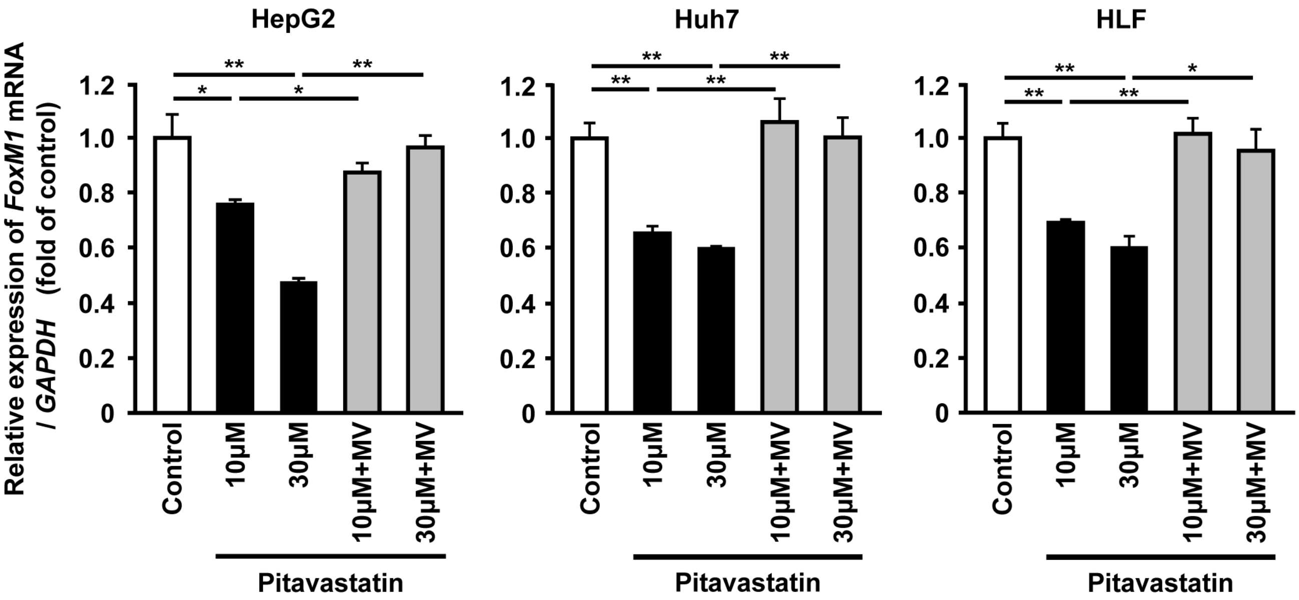

Supplementary Figure 1: *FoxM1* mRNA expression is regulated by the mevalonate pathway in human hepatoma cells. Quantitative real time RT-PCR analysis showing the gene expression of *FoxM1* in HepG2 cells (left panel), Huh7 cells (middle panel), and HLF cells (right panel) after treatment with pitavastatin (10 μM or 30 μM), either alone or along with MV (100 μM), for 24 hours. Data are expressed as mean ± SEM, $^{*}p < 0.05$, $^{**}p < 0.01$.

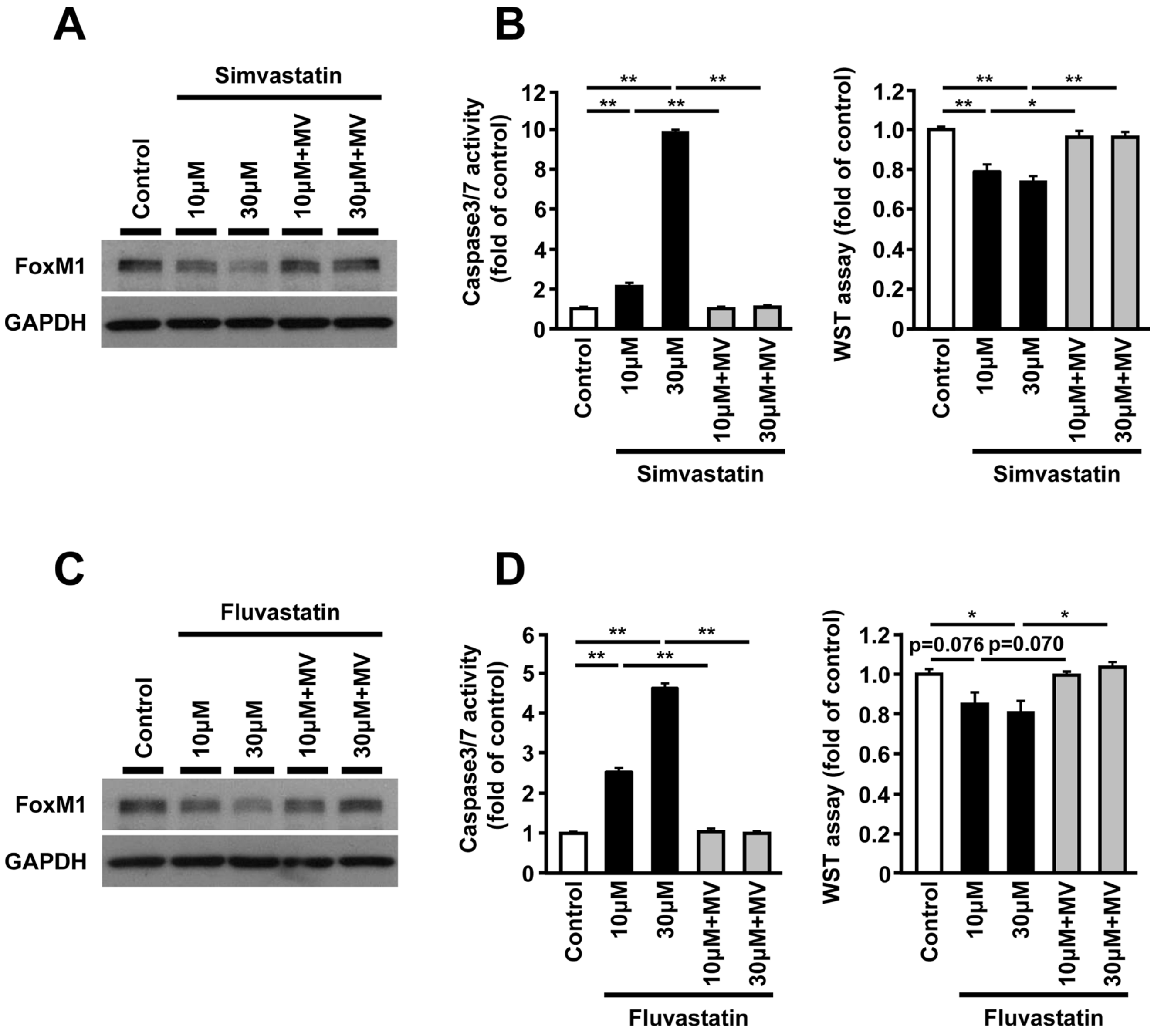

Supplementary Figure 2: Simvastatin or fluvastatin has similar effects to pitavastatin on FoxM1 expression and cell death in human hepatoma cells. (**A**) Western blot analysis showing the protein expression of FoxM1 in HepG2 cells treated with simvastatin (10 μM or 30 μM), either alone or along with MV (100 μM). (**B**) Assessment of cell death by caspase3/7 activity (left panel) or cell viability by WST assay (right panel) treated with simvastatin (10 μM or 30 μM), either alone or along with MV (100 μM). (**C**) Western blot analysis showing the protein expression of FoxM1 in HepG2 cells treated with fluvastatin (10 μM or 30 μM), either alone or along with MV (100 μM). (**D**) Assessment of cell death by caspase3/7 activity (left panel) or cell viability by WST assay (right panel) treated with fluvastatin (10 μM or 30 μM), either alone or along with MV (100 μM). Data are expressed as mean ± SEM, $^{*}p < 0.05$, $^{**}p < 0.01$.

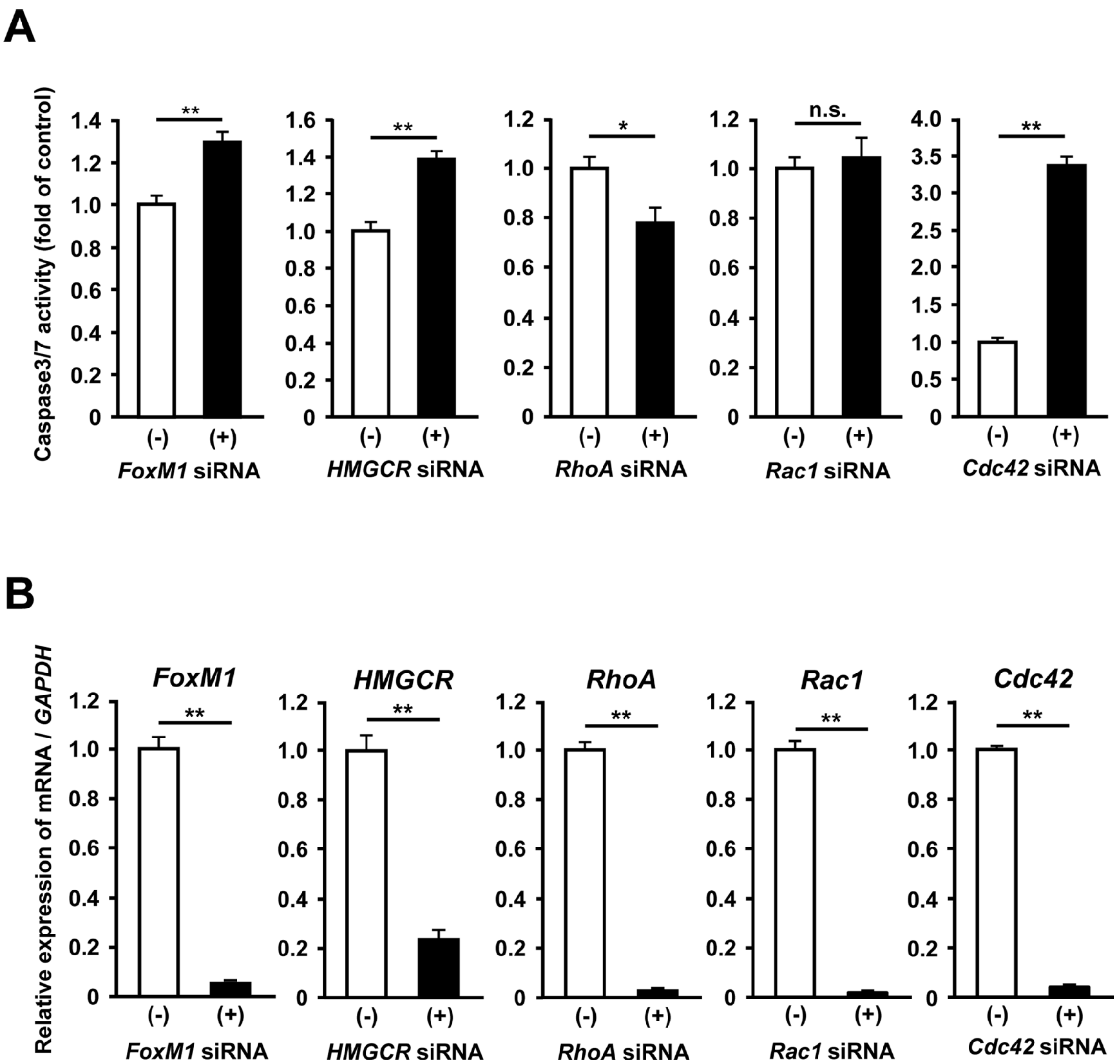

Supplementary Figure 3: The knockdown of *FoxM1*, *HMGCR*, or *Cdc42* but *RhoA*, or *Rac1*, resulted in cell death of human hepatoma cells. (**A**) Effect of siRNA-mediated depletion of *FoxM1*, *HMGCR*, *RhoA*, *Rac1*, or *Cdc42* on cell death of HepG2 cells assessed by caspase3/7 activity. (**B**) Quantitative real-time RT-PCR analysis showing the gene expressions of *FoxM1*, *HMGCR*, *RhoA*, *Rac1*, or *Cdc42* in siRNA-treated HepG2 cells. Data are expressed as mean ± SEM, $^{*}p < 0.05$, $^{**}p < 0.01$, n.s. not significant.

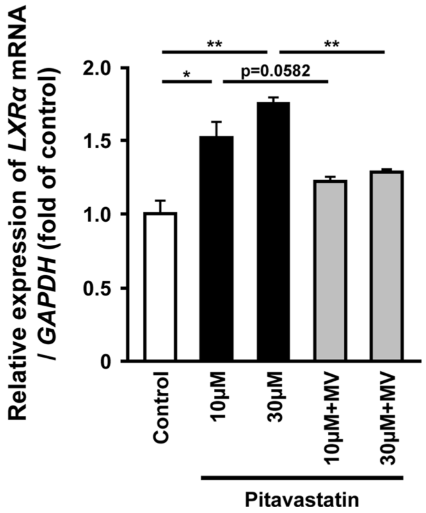

Supplementary Figure 4: *LXRα* mRNA expression is regulated by the mevalonate pathway in human hepatoma cells. Quantitative real time RT-PCR analysis showing the gene expression of *LXRα* in HepG2 cells after treatment with pitavastatin (10 µM or 30 µM), either alone or along with MV (100 µM), for 24 hours. Data are expressed as mean ± SEM, $^{*}p < 0.05$, $^{**}p < 0.01$.

Supplementary Table 1: Patients' characteristics according to *FoxM1* and *HMGCR* expression in the tumor tissues of HCC patients

	***FoxM1*- and *HMGCR*-high (*n* = 16)**	***FoxM1*- and/or *HMGCR*-low (*n* = 48)**	***p*-value**
**Age (y.o.), mean (range)**	66.7 (47–84)	63.4 (36–84)	0.3596
**Gender, Male, *n* (%)**	15 (93.8)	42 (87.5)	0.4879
**HBs-Ag, Positive, *n* (%)**	3 (18.8)	12 (25.0)	0.6093
**HCV-Ab, Positive, *n* (%)**	11 (68.8)	22 (45.8)	0.1122
**Child-Pugh score, A, *n* (%)**	16 (100)	41 (85.4)	0.1055
**AFP (ng/ml), median (range)**	29.5 (5–390000)	15.0 (3–193800)	0.4053
**PIVKA-II (mAU/ml), median (range)**	1402.5 (40–102288)	114.5 (13–361200)	0.0529
**Liver histology, LC, *n* (%)**	1 (6.3)	16 (33.3)	0.0337
**Maximum tumor size (mm), mean (range)**	47.3 (17–150)	43.4 (7–320)	0.3978

LC, liver cirrhosis.

Supplementary Table 2: Patients' characteristics according to *FoxM1* and *SREBP2* expression in the tumor tissues of HCC patients

	FoxM1- and *SREBP2*-high (n = 18)	*FoxM1*- and/or *SREBP2*-low (n = 46)	p-value
**Age (y.o.), mean (range)**	63.8 (49–82)	64.4 (36–84)	0.7253
**Gender, Male, *n* (%)**	16 (88.9)	41 (89.1)	0.9778
**HBs-Ag, Positive, *n* (%)**	6 (33.3)	9 (19.6)	0.2424
**HCV-Ab, Positive, *n* (%)**	9 (50.0)	24 (52.1)	0.8757
**Child-Pugh score, A, *n* (%)**	17 (94.4)	40 (87.0)	0.3882
**AFP (ng/ml), median (range)**	39 (5–390000)	15 (3–193800)	0.2307
**PIVKA-II (mAU/ml), median (range)**	1122 (40–205074)	114.5 (13–361200)	0.0294
**Liver histology, LC, *n* (%)**	4 (22.2)	13 (28.3)	0.6229
**Maximum tumor size (mm), mean (range)**	51.1 (17–150)	41.8 (7–320)	0.2349

LC, liver cirrhosis.

Supplementary Table 3: Factors affecting overall survival of HCC patients

			Univariate	Multivariate		
		n = 64	p-value	HR	95% CI	p-value
**Age (y.o.)**	**≥67**	32	0.4958			
	**<67**	32				
**Gender**	**Male**	57	0.3160			
	**Female**	7				
**HBs-Ag**	**Negative**	49	0.2538			
	**Positive**	15				
**HCV-Ab**	**Negative**	31	0.3210			
	**Positive**	33				
**Child-Pugh score**	**A**	57	0.7696			
	**B**	7				
**AFP (ng/ml)**	**≥20**	32	0.9819			
	**<20**	32				
**PIVKA-II (mAU/ml)**	**≥300**	32	0.0070	2.72	0.84–10.6	0.0974
	**<300**	32		1		
**Liver histology**	**LC**	17	0.1172			
	**non LC**	47				
**Maximum tumor size (mm)**	**≥33**	32	0.0436	1.98	0.67–6.47	0.2193
	**<33**	32		1		
**mRNA expression in HCC**	***FoxM1*- and *HMGCR*-high**	16	0.0591	2.38	0.84–6.47	0.1002
	***FoxM1*- and/or *HMGCR*-low**	48		1		

LC, liver cirrhosis; HR, hazard ratio; CI, confidence interval.

Supplementary Table 4: Factors affecting overall survival of HCC patients

			Univariate	Multivariate		
		n = 64	*p*-value	HR	95% CI	*p*-value
**Age (y.o.)**	**≥67**	32	0.4958			
	**<67**	32				
**Gender**	**Male**	57	0.3160			
	**Female**	7				
**HBs-Ag**	**Negative**	49	0.2538			
	**Positive**	15				
**HCV-Ab**	**Negative**	31	0.3210			
	**Positive**	33				
**Child-Pugh score**	**A**	57	0.7696			
	**B**	7				
**AFP (ng/ml)**	**≥20**	32	0.9819			
	**<20**	32				
**PIVKA-II (mAU/ml)**	**≥300**	32	0.0070	2.63	0.80–10.3	0.1127
	**<300**	32		1		
**Liver histology**	**LC**	17	0.1172			
	**non LC**	47				
**Maximum tumor size (mm)**	**≥33**	32	0.0436	2.00	0.68–6.60	0.2137
	**<33**	32		1		
**mRNA expression in HCC**	***FoxM1*- and *SREBP2*-high**	18	0.0131	3.24	1.18–8.89	0.0238
	***FoxM1*- and/or *SREBP2*-low**	46		1		

LC, liver cirrhosis; HR, hazard ratio; CI, confidence interval.
